# Supplementary material for: Are elevated plasma fibrinogen associated with lung function? An 8-year follow-up of the ELSA study
Source: PLoS One. 2021 Nov 8;16(11):e0259498. doi: 10.1371/journal.pone.0259498 (PMC8575306; doi:10.1371/journal.pone.0259498)
Supplement: Attachment — Submitted filename: Review comments.pdf [file pone.0259498.s001.pdf]

Review comments

PONE-D-20-39275
Are elevated plasma fibrinogen associated with lung function? An 8-year follow-up of the ELSA study

Overall Impressions

This is an important scientific work that brings out the correlation between the level of fibrinogen and lung functions in old men. From the data and conclusion of the authors, significantly high levels of plasma fibrinogen are observed in the old men with lung problem. Thus the implementation of this parameter as a biomarker or indicator in the diagnosis and/or prognostics of lung dysfunction could be envisaged after a thorough follow up or control of this work.

Corrections

Minor

Page 2, Line 28. 'percentages of predicted' should read 'percentages predicted'

Line 33. **Conclusion: '**We found' should read 'It was found'

Line 37. **Key words:** 'Respiratory Function Tests' should read 'Respiratory Functions'

Page 4, Line 88. 'plasma is dispensed' should read 'plasma was dispensed'

Line 88. 'C XL reagent is added' should read 'C XL reagent was added'

Page 5, Line 103. 'FEV1 and FVC expressed' should read 'FEV1 and FVC were expressed'

Page 7, Line 150. Table 1 ………. 'ELSA who presented complete data from for fibrinogen'

Major

None detected

CONCLUSION

Publication recommended after the minor revisions outlined above.
