# Supplementary material for: Are elevated plasma fibrinogen associated with lung function? An 8-year follow-up of the ELSA study
Source: PLoS One. 2021 Nov 8;16(11):e0259498. doi: 10.1371/journal.pone.0259498 (PMC8575306; doi:10.1371/journal.pone.0259498)
Supplement: Attachment — Submitted filename: review.docx [file pone.0259498.s002.docx]

Thank you for inviting me to review the manuscript entitled **“**Are elevated plasma fibrinogen associated with lung function? An 8-year follow-up of the ELSA study**”** It is a good piece of work however, in my opinion it lacks novelty.
