# Supplementary material for: Are elevated plasma fibrinogen associated with lung function? An 8-year follow-up of the ELSA study
Source: PLoS One. 2021 Nov 8;16(11):e0259498. doi: 10.1371/journal.pone.0259498 (PMC8575306; doi:10.1371/journal.pone.0259498)
Supplement: Attachment — Submitted filename: Response to Reviewers.docx [file pone.0259498.s003.docx]

Dear Emiliano Giardina and Reviewers,

We would appreciate the consideration of our Research Article entitled “Are elevated fibrinogen plasma levels associated with lung function? An 8-year follow-up of the ELSA study” for publication in PLOS One.

Thank you for the reviewers' considerations and suggestions. After corrections we present a new version of the article, and we hope to be in agreement for publication.

Journal Requirements:

When submitting your revision, we need you to address these additional requirements.

**1. Please ensure that your manuscript meets PLOS ONE's style requirements, including those for file naming. The PLOS ONE style templates can be found at**

<https://journals.plos.org/plosone/s/file?id=wjVg/PLOSOne_formatting_sample_main_body.pdf> and

<https://journals.plos.org/plosone/s/file?id=ba62/PLOSOne_formatting_sample_title_authors_affiliations.pdf>

We appreciated this comment, and we review the style of the document.

**2. In the methods section please provide details regarding how house hold wealth and physical exercise was stratified for analysis.**

Thanks for the comment. We added at the references that describle how these variables are stratified.

*Socioeconomic position was based on the National Statistics Socio-Economic Classification (NS-SEC) and quintiles of total wealth, defined from the sum of financial, physical (e.g. businesses, land) and housing wealth, minus debts and pension payments (Banks J, Batty G, Coughlin K, et al (2019) English Longitudinal Study of Ageing: Waves 0-8, 1998-2017. 29th Edition. UK Data Service. SN: 5050, London)*

*Participants were asked how often they took part in three different types of physical activity: vigorous, moderate and low intensity. The response options were more than once a week, once a week, one to three times a month and hardly ever/never. For the purposes of the analysis, we derived a summary index of physical activity by summing responses to the three physical activity questions after they had been dichotomised around the frequency cut-point of once a week or more often. The derived summary index categorised physical activity as follows: (1) physical inactivity; (2) low-intensity but not vigorous/moderate-intensity physical activity at least once a week; and (3) vigorous/moderate-intensity physical activity at least once a week. (Demakakos, P., Hamer, M., Stamatakis, E. et al. Low-intensity physical activity is associated with reduced risk of incident type 2 diabetes in older adults: evidence from the English Longitudinal Study of Ageing. Diabetologia* ***53,*** *1877–1885 (2010). https://doi.org/10.1007/s00125-010-1785-x)*

**3. Thank you for stating the following in the Acknowledgments Section of your manuscript:**

**"The English Longitudinal Study of Ageing was developed by a team of researchers based at the University College London, NatCen Social Research, the Institute for Fiscal Studies and the University of Manchester. The NatCen Social Research collected the data. The National Institute of Aging Grant R01AG017644 and a consortium of UK government departments coordinated by the Economic and Social Research Council provide the funding."**

**We note that you have provided funding information that is not currently declared in your Funding Statement. However, funding information should not appear in the Acknowledgments section or other areas of your manuscript. We will only publish funding information present in the Funding Statement section of the online submission form.**

**Please remove any funding-related text from the manuscript and let us know how you would like to update your Funding Statement. Currently, your Funding Statement reads as follows:**

**"Funders did not participate in the study design, data collection and analysis, decision**
**to publish or prepare the manuscript."**

**Please include your amended statements within your cover letter; we will change the online submission form on your behalf.**

Thanks for the corrections, all were executed in the manuscript.

**4. We note that you have indicated that data from this study are available upon request. PLOS only allows data to be available upon request if there are legal or ethical restrictions on sharing data publicly. For more information on unacceptable data access restrictions, please see** [**http://journals.plos.org/plosone/s/data-availability#loc-unacceptable-data-access-restrictions**](http://journals.plos.org/plosone/s/data-availability#loc-unacceptable-data-access-restrictions)**.**

**In your revised cover letter, please address the following prompts:**

**a) If there are ethical or legal restrictions on sharing a de-identified data set, please explain them in detail (e.g., data contain potentially sensitive information, data are owned by a third-party organization, etc.) and who has imposed them (e.g., an ethics committee). Please also provide contact information for a data access committee, ethics committee, or other institutional body to which data requests may be sent.**

**b) If there are no restrictions, please upload the minimal anonymized data set necessary to replicate your study findings as either Supporting Information files or to a stable, public repository and provide us with the relevant URLs, DOIs, or accession numbers. For a list of acceptable repositories, please see** [**http://journals.plos.org/plosone/s/data-availability#loc-recommended-repositories**](http://journals.plos.org/plosone/s/data-availability#loc-recommended-repositories)**.**

We will update your Data Availability statement on your behalf to reflect the information you provide.

**Data Sharing**

The English Longitudinal Study of Ageing data are available to the scientific community from the UK Data Service for researchers who meet the criteria for access to confidential data, under conditions of the End User License <http://ukdataservice.ac.uk/media/455131/cd137-enduserlicence.pdf>. The data can be accessed from: <https://beta.ukdataservice.ac.uk/datacatalogue/series/series?id=200011#!/access-data>. Contact with the UK Data Service regarding access to the English Longitudinal Study of Ageing can be made through the website <https://www.ukdataservice.ac.uk/about-us/contact>, by phone +44 (0)1206 872143 or by email at [help@ukdataservice.ac.uk](mailto:help@ukdataservice.ac.uk).

**5. PLOS requires an ORCID iD for the corresponding author in Editorial Manager on papers submitted after December 6th, 2016. Please ensure that you have an ORCID iD and that it is validated in Editorial Manager. To do this, go to ‘Update my Information’ (in the upper left-hand corner of the main menu), and click on the Fetch/Validate link next to the ORCID field. This will take you to the ORCID site and allow you to create a new iD or authenticate a pre-existing iD in Editorial Manager. Please see the following video for instructions on linking an ORCID iD to your Editorial Manager account:** [**https://www.youtube.com/watch?v=_xcclfuvtxQ**](https://www.youtube.com/watch?v=_xcclfuvtxQ)

Thanks for the comments. The author Ione Schneider has two accounts in PLOS. One is related to username ione.schneider and the other one, to the username ione.jayce. We need to merge these two accounts to be able to validate with Ione’s ORCID (0000-0001-6339-7832).

**6. Your ethics statement should only appear in the Methods section of your manuscript. If your ethics statement is written in any section besides the Methods, please delete it from any other section.**

The paragraph on ethics statement was removed from the end and is only described in the methods section.

Reviewer #1:

**Corrections**

**Page 2, Line 28. ‘percentages of predicted’ should read ‘percentages predicted’**
**Line 33. Conclusion: ‘We found’ should read ‘It was found’**
**Line 37. Key words: ‘Respiratory Function Tests’ should read ‘Respiratory Functions’**
**Page 4, Line 88. ‘plasma is dispensed’ should read ‘plasma was dispensed’**
**Line 88. ‘C XL reagent is added’ should read ‘C XL reagent was added’**
**Page 5, Line 103. ‘FEV1 and FVC expressed’ should read ‘FEV1 and FVC were expressed’**
**Page 7, Line 150. Table 1 ………. ‘ELSA who presented complete data from for fibrinogen’**

Thanks for the corrections, all were executed in the manuscript.

**Reviewer #2:**

**Thank you for inviting me to review the manuscript entitled “Are elevated plasma fibrinogen associated with lung function? An 8-year follow-up of the ELSA study” It is a good piece of work however, in my opinion it lacks novelty.**

Thanks for the comments. This is the first time that the relation between lung function and fibrinogen is studied in the English population.

**Reviewer #3:**

**In this study, the authors reported that a high level of plasma fibrinogen in older English men, but not women, is associated with a decline in lung function. In my opinion, the study is interesting however, I have some major concerns regarding the study design and the inclusion criteria implied in this study. I would like the authors to address the following concerns;**
**1- The major concern is the inclusion of subjects with pulmonary diseases which affect the lung function in these subjects and thus affect the reliability of the results. These subjects should be excluded to express only the relationship between fibrinogen level and lung function.**

The questionnaire used contained information about self-reported lung diseases (COPD and asthma). Thus, this self-reported information was used as adjustment variables in the analyses and interactions and multicollinearity between variables were tested, which were not detected.

**2- It is not clear is there any subjects in this study with cardiocirculatiry disorder?**

The disease high blood pressure, angina, heart attack, congestive heart failure, heart murmur, and abnormal heart rhythm were classified in the presence of one or more. However, they were excluded due to multicollinearity.

**3- The study design regarding the timeline of fibrinogen assessment and Lung function evaluation should be further described briefly. What is meant by wave 2, 4, and 6? Please provide a brief clear description of this part in methodology.**

Thanks for the comments. We clarified the information about the timeline of fibrinogen and lung function between the waves.

**4- Methods: Line 80: “those with a clotting or bleeding disorder or on anticoagulant drugs, those who had ever had…. were not asked to fast.” These cases were stated to be excluded. In this statement they were asked not to fast before sampling? Did you include these cases?**

The members with described problems are not eligible to the blood sample. This way, these cases are not included in the analysis.

**5- In table 2: the significance should be defined with p-values.**

This information was updated at the table.

**6- The limitations: Selection bias should be addressed as a limitation in this study.**

Thanks for the comments. We addressed the selection bias to the limitations.

Thank you for your time and consideration.

Yours sincerely

Dr Ione Jayce Ceola Schneider

Affiliate Academic

University College London, Department
